# Antidiabetic Medicines Utilisation During Pre-Pandemic, Pandemic and Post-Pandemic Period of COVID-19—Data for Bulgarian Population

**DOI:** 10.3390/healthcare13030322

**Published:** 2025-02-04

**Authors:** Zornitsa Mitkova, Desislava Stanimirova, Miglena Manova, Nikolay Gerasimov, Konstantin Mitov, Guenka Petrova

**Affiliations:** 1 Department of Organization and Economy of Pharmacy, Faculty of Pharmacy, Medical University-Sofia, 1000 Sofia, Bulgaria; desi_stanimirova@abv.bg (D.S.); miglenamanova@yahoo.com (M.M.); kmitov@pharmfac.mu-sofia.bg (K.M.); guenka.petrova@gmail.com (G.P.); 2 Medical College, Trakia University, 6000 Stara Zagora, Bulgaria; nik.gerasimov.phd@gmail.com

**Keywords:** antidiabetics, utilisation, DDD/1000 inh/day, public expenditure, cost dynamics

## Abstract

Background: Type 2 diabetes is a chronic disease with high global prevalence and significant social and economic burden. The pandemic affected patients’ diagnostics and medicines dispensing. Diabetes was among the most-affected conditions during lockdown due to the limited resources and unaffordable medicines. The impact of the pandemic on utilisation and cost has not been thoroughly studied, which inspired us to conduct the current study. Objectives: The study explored cost dynamics, changes in antidiabetic medicines utilisation, and public expenditure of pharmacotherapy in three periods: pre-pandemic (2018–2019), during the pandemic (2020–2021), and post-pandemic (2022–2023). Methods: It is a retrospective, observational, macroeconomic analysis. Reimbursed cost and utilisation were analysed as a crude sum and as indexes of the average value. Results: The result shows that five new INNs have been included in the Positive Medicines List (PML), two of these being fixed dose combinations (FDCs). During the pandemic, a slow tendency of increase of the crude sum of public expenditure was observed, followed by a sharp increase in the post-pandemic period. The public spending increased more than twice, and we found a 30,018,982 Euro growth. The highest public spending is found for dapagliflozine in post-pandemic vs. pandemic period (index = 1.67), as well as empagliflozin/metformin and dapagliflozine in pandemic vs. pre-pandemic period (index = 0.21). Total utilisation increases from 58.16 to 71.78 DDD/1000 inh/day during 2018–2023. The most significant rise of utilisation is found for canagliflozin (index = 0.68) pandemic vs. pre-pandemic and dapagliflozin (index = 3.66) post-pandemic vs. pandemic. Conclusions: Analysis of the antidiabetic medicines market reveals the rising of reimbursed cost and utilisation in pre-, post-, and during the pandemic. In conclusion, organisation of the supply and financing of antidiabetic medicines was not affected during the pandemic.

## 1. Introduction

Diabetes is a chronic disease, an important public health problem, and one of four priority non-communicable diseases (NCDs) targeted for action by health authorities worldwide [1].The global prevalence of Type 2 diabetes has increased, particularly in low- and middle-income countries (LMICs). Related co-morbidities and complications can deteriorate patient health and increase costs. In the case of diabetes, it is common that direct costs generally exceed indirect costs [2], with values of 76.2% (US$28.73 per patient) for direct and 23.8% (US$9.50 per patient) for indirect costs [3,4]. In the case of additional circumstances such as influenza or other infectious diseases like COVID-19, the cost and health-related quality of life of diabetic patients can further deteriorate.

Bulgaria is not exempt from the global rise of diabetes and the associated costs. With approximately 28,000 individuals diagnosed with Type 1 diabetes and 452,490 with Type 2 aged between 60 and 74 years, the cost and social burden are heavy [5,6].

Timely and affordable therapy is crucial for diabetics to cope with the disease and to decrease individual and societal costs. The disease management requires regular glucose monitoring and adherence to medical recommendations regarding lifestyle measures and assessment of the risk of complications [7]. The literature review confirmed that diabetes was the most affected condition during lockdown due to the limited resources, unaffordable medicine, delayed care, transport difficulties, and undiagnosed cases, etc. [8]. During the COVID-19 pandemic, many countries noted drug shortages and suffered from broken supply chains [9]. The shortages were recorded not only for medicines used for COVID-19 but also for intensive care units, for patients with chronic diseases and complications. Urgent efforts were made to find optimal medical products for prevention, diagnostics, and treatment during COVID-19, as well as to avoid falsified medicines. Manufacturing and supply chains of many key medical products, including essential products, were a concern during this crisis [10]. Measures for export bans and new licensing requirements significantly affected medicine availability and patients’ affordability [11].

The literature searches reveal that the pandemic’s impact on utilisation and cost of diabetes therapy has not been studied thoroughly worldwide. This inspired us to conduct the current study and basically to assess how the pandemic affected diabetes in Bulgaria. The aim of this study is to explore market dynamics, changes in the utilisation, and public expenditure on pharmacotherapy of per oral antidiabetic medicines in Bulgaria during the pre-pandemic, pandemic, and post-pandemic periods.

## 2. Materials and Methods

### 2.1. Design of the Study

This study is a retrospective, observational, macroeconomic analysis of public expenditure on the main groups of antidiabetic medicines reimbursed in Bulgaria. An object of observation was per oral medicines from Anatomical Therapeutic Chemical (ATC) codes: A10BF01, A10BX11, A10BD16, A10BX09, A10BD15, A10BX12, A10BD19, A10BD20, A10BB09, A10BB12, A10BH05, A10BD11, A10BA02, A10BG03, A10BD05, A10BX02, A10BD10, A10BD21, A10BJ06, A10BH01, A10BD07, A10BH02, and A10BD08.

A three-step analysis of market dynamics is performed. First, the new products that entered the market are presented, then the public costs related to antidiabetic medicines are calculated, and finally the utilisation of the medicinal products was calculated as DDD/1000 inh/day. The study encompasses three periods: pre-pandemic (2018–2019); pandemic (2020–2021), and post-pandemic (2022–2023). Therefore, the utilisation, spending, and penetration of new medicines is compared between the three periods in order to estimate the supply, availability, and affordability of antidiabetics.

### 2.2. Data Sources

The National Health Insurance Fund (NHIF) database was used to extract information about the reimbursed cost and the number of dispensed packages for every peroral antidiabetic medicine. Cost and quantities were then grouped per International non-proprietary name (INN) on an annual basis [12]. Costs are presented in Euros with the exchange rate BGN 1 = EUR 0.5113 [13].

The National Statistical Institute (NSI) database provides information about the population for every year in consideration (2018–2023) [14].

The National Council of Prices and Reimbursement of Medicines database was searched for the new per oral antidiabetic medicines introduced in the years of observation [15].

### 2.3. Market Dynamic Analysis

All newly included antidiabetic medicines in the Positive Drug List (PDL) during the observed period were extracted on a yearly basis, and their introduction in the treatment practice was analysed based on the reimbursed cost and utilisation in defined daily doses (DDD)/1000 inh/day (DTD).

### 2.4. Cost Analysis

Data on the reimbursed quantities and sum paid by the NHIF were summarised for every year of observation per INN [9]. Reimbursed costs (RCs) are compared between individual INNs during 2018–2023 and between total reimbursed costs per year. The reimbursed cost was analysed as a crude sum and as indexes of average value changed between the three observed periods via the formula:

Cost index (pandemic vs. pre-pandemic period) = (((RC(2020 + 2021)/2) − (RC (2018 + 2019))/2)/((RC(2018 + 2019)/2))

and

Cost index (post pandemic vs. pandemic period) = (((RC(2022 + 2023)/2) − (RC (2020 + 2021))/2)/((RC(2020 + 2021)/2))

### 2.5. Utilisation Analysis

The World Health Organization (WHO) methodology was used to estimate the utilisation in DDD per 1000 inhabitants per day (DTD) [16].

$$\mathrm{DTD}=\frac{\left(\mathrm{Sales}\;\mathrm{in}\;\mathrm{mg}/\mathrm{DDDmg}\right)/}{\left(\mathrm{Number}\;\mathrm{of}\;\mathrm{inhabitants}\times 365\right)}\times 1000$$

Changes in the utilisation in DDD/1000 ind/day were analysed per INN, for the whole group of per oral antidiabetic and as indexes of change using the formulas:

Utilisation Index (pandemic vs. pre-pandemic period) = (((DTD (2020 + 2021)/2) − (DTD (2018 + 2019))/2)/((DTD (2018 + 2019)/2))

and

Utilisation Index (post-pandemic vs. pandemic period) = (((DTD (2022 + 2023)/2) − (DTD (2020 + 2021))/2)/((DTD (2020 + 2021)/2))

### 2.6. Ethical Aspects

The information for the study is gathered from publicly available databases with reimbursed cost and utilized quantities of medicines and not from individual patients. Therefore, we do not apply for ethical approval. Publicly available sources of information are cited in the reference list.

## 3. Results

### 3.1. Market Dynamic Analysis

Five new INNs were included in the PDL during 2018–2023. Semaglutide is the only monoproduct included in 2022. The following fixed dose combinations (FDC) were included—canagliflozin/metformin in 2019; pioglitazone/metformin in 2019; empagliflozin/linagliptin in 2020; andsaxagliptin/dapagliflozin in 2021. The fact that two of the FDCs were included in the PDL during the COVID-19 pandemic (2020–2021) might be commented as positive regulatory efforts to ensure new therapies, even during the pandemic.

The FDC of canagliflozin/metformin has decreased its cost to the public payer with a negative index of −0.06 (6% decrease) after a COVID-19 period, in comparison with the pandemic period (Table 1). Oppositely, the FDC empagliflozin/linagliptin has increased its reimbursement cost steadily from 0.4 to 1.4 million Euro and with 68% after the pandemic. The other two FDC increased their reimbursed cost with almost three times higher expenditures (saxagliptine/metformin) and a positive index of 15% after the pandemic (pioglitason/metformin). The cost tendencies match the utilisation in DTD for all newly introduced medicines. We can consider that the payer tried to ensure the necessary financial means for treatment of patients with the new peroral antidiabetic medicines.

### 3.2. Cost Analysis

Table 1 presents the crude reimbursed cost and index of cost change during the three observed periods.

**Figure 1:**
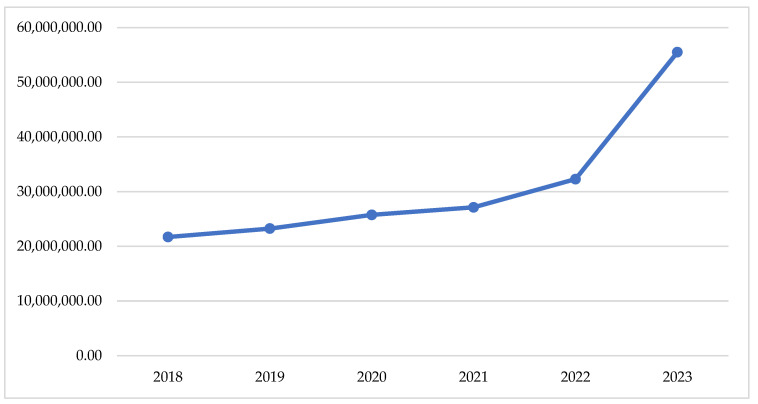
Total reimbursed cost during the observed period.

If we look at the changes in the total reimbursed costs, we can find a slow tendency of increase of the crude sum during the pandemic period (2020–2021) and a sharp increase after that (2022–2023)—Figure 1. This observation is supported by the total index of 0.04, comparing the pandemic with the pre-pandemic period and of 0.17, comparing the post-pandemic with the pandemic (Table 1).

**Figure 2:**
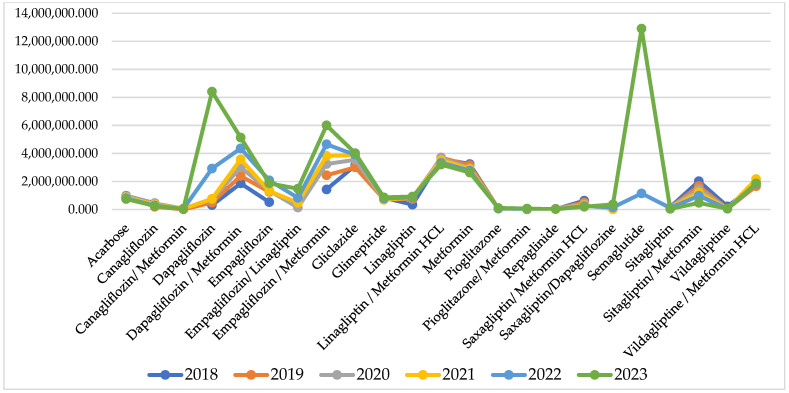
Reimbursed cost per INN.

For some of the INNs, reimbursed cost sharply increased after the pandemic, but for many of them, it remains similar to the pandemic and pre-pandemic period (Figure 2).

Comparing the index of cost changes, 9 of the INNs have negative indexes, varying between −0.01 to −0.16 and showing that for those INNs, the reimbursed cost decreased from 2% to 16%. After the pandemic, there are 10 INNs with negative indexes of cost change varying between −0.02 to −0.11. Out of those 10 INNs, 6 are the same molecules as from the pandemic period, while the rest are different (Table 1).

The highest costs are observed for therapy with semaglutide (12,908,341 Euro) dapagliflozin/metformin (5,136,999 Euro), empagliflozin/metformin (6,001,390 Euro), and dapagliflozin/metformin (5,136,999 Euro). Overall, NHIF costs for peroral antidiabetes therapy are rising every year and for the investigated period we found a 30,018,982 Euro growth.

### 3.3. Utilisation Analysis

**Figure 3:**
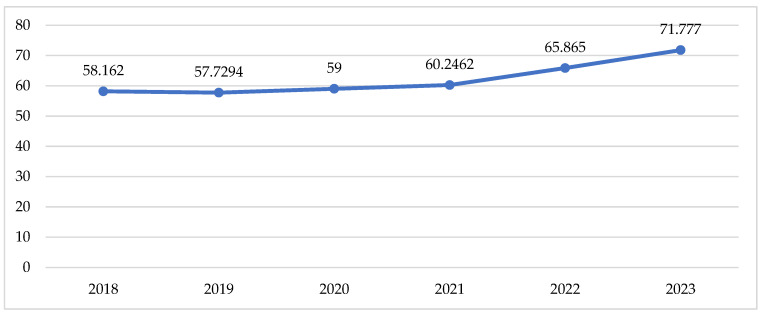
Utilisation of peroral antidiabetic in DTD.

Total utilisation in DTD follows the tendency described above for the cost data, with a slow increase during the pandemic and a sharp increase in the post-pandemic period (Table 2, Figure 3).

**Figure 4:**
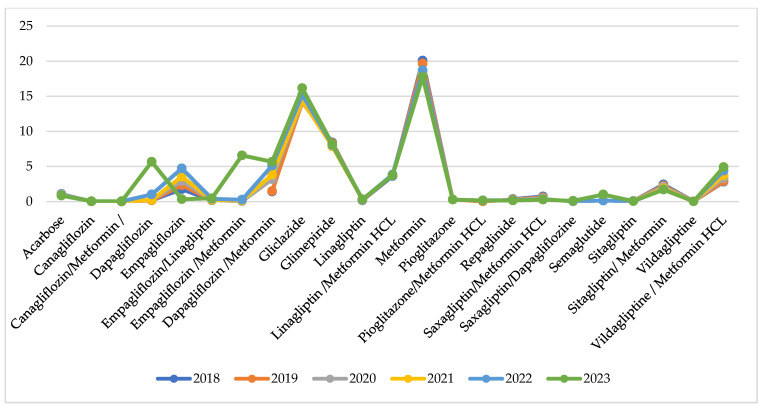
Utilisation in DTD per INN.

Utilisation per INN in DTD is similar during the observed years with no extreme fluctuations for the INNs under consideration (Figure 4).

Indexes of total utilisation are positive and have increased from 0.01 to 0.04 for the compared periods (Table 2). This might be commented as growth in utilisation after the pandemic. Comparing the pandemic with the pre-pandemic period, we observe 8 INNs with negative indexes of utilisation varying from −0.01 to −0.09 (from 1 to 9% decrease in utilisation). During the post-pandemic period, 9 INNs decreased their utilisation index from −0.01 to −0.08, meaning that the crude utilisation of INN is declining. Only 5 INNs with decreasing indexes of utilisation are the same as in the previous period (Table 2).

## 4. Discussion

To the best of our knowledge, this is the first study exploring per oral antidiabetics reimbursed cost and utilisation during pre-, post-, and pandemic periods in Bulgaria. We focus on the antidiabetic medicines because other studies already proved that there is an association between antidiabetic drugs and COVID-19. SGLT-2 and metformin appears to be protective against COVID-19 hospitalization and infection; repaglinide against infection. On the other side, insulin and sulfonylureas appears to be risk factors for COVID-19 hospitalization and infection [17]. Therefore, any supply shortages of antidiabetic medicines potentially could deteriorate diabetes.

In this study, we aimed to investigate whether the COVID-19 pandemic had a negative impact on diabetic patients in terms of ensuring medicines and the financial resources needed. The results, in general, are encouraging. From one side, we can see that even during the pandemic period (2020–2021),two new INNs were included in the treatment armamentarium against Type 2 diabetes. The total reimbursed cost and utilisation are increasing in crude values and as indexes that we might also consider as encouraging results. The fact that post-pandemic cost and utilisation are increasing sharply could be commented as probable, reserved, and conservative behaviour from payers, physicians, and patients during the pandemic, but further studies are needed to confirm such a suggestion. We also observed that on an individual INN level, cost and utilisation are varying in both explored indicators, which might be attributed to company policies and physician preferences, but there is no evidence that can confirm such a suggestion.

In other countries’ drug shortages, low hospitalisation rates and limited physicians’ visits are often reported during the pandemic, as well as noncompliance from the patients’ side, which leads to worsening symptoms of chronic diseases [18,19].

During the pandemic, reductions in outpatient visits and testing was observed, compared with 2019, while the prescriptions fill was almost the same (64.2% vs. 62.2%; 3.6%) in USA [20]. At the same time, diabetic patients need special attention and use essential healthcare resources. [21,22] The costs for therapy among people with Type 2 diabetes in good glycaemic control is significantly lower (EUR 25,018) than those among patients with poor glycaemic control (EUR 57,244) during the first wave of COVID-19 in Europe [23]. As observed in our study as well, at the beginning of the pandemic, in Sweden, a decline was found for each initiation of antidiabetic drug treatment, but the level of utilisation increased quickly thereafter [24]. Our study reveals that total annual utilisation of antidiabetics in Bulgaria increased each year, in comparison with the previous one. The most significant increase was observed for canagliflozin during the pandemic vs. pre-pandemic period (index = 0.68) and dapagliflozin levels in the post-pandemic vs. pandemic period (index = 3.66).

At a general level, we do not observe treatment interruption, which was reported in France and the UK, as well as a decline in treatment initiation [25,26].The study in Greece reveals that antidiabetics utilisation decline compared between 2019 and 2020 from 34,150 g day^−1^ in 2019 to 13,014 g day^−1^ in 2020, affected by lockdown and pandemic [27]. The total treatment cost and utilisation of per oral antidiabetics in our study follow similar tendencies, with small growth during the pandemic and a sharp increase after that. In Turkey, a significant increase in the utilisation and expenditure of antidiabetic drugs during the COVID-19 pandemic was found, compared with the pre-pandemic period [28]. We also found that in Bulgaria, public spending on antidiabetic medicines is increasing annually, but the difference between 2018 and 2023 is very impressive, as the cost rose more than twice. We found as low increase in the crude sum during the pandemic period (2020–2021) and a sharp increase after that (2022–2023). We found that the highest is the increase of public expenditures for dapagliflozine in the post-pandemic vs. pandemic period (index = 1.67), as well as both empagliflozin/metformin and dapagliflozine in the pandemic vs. pre-pandemic period (index = 0.21). The public spending for medicines during the COVID-19 pandemic in Tanzania remained similar, but in Kenya, it declined significantly [29].

The lack of similar studies makes for a difficult comparison of the results from our study. Further studies are needed to estimate the impact of the COVID-19 pandemic on both medicines utilisation and public expenditures. The assessment of therapy initiation is very important during the lockdown, especially those who were hindered due to limited resources, as it can present the gaps of the healthcare system. The public spending variations also reveal that during the pandemic, some fluctuations in medicines prescribing, dispensing, or utilisation could exist. The avoidance of hospitalizations due to diabetes during the pandemic declined from 17.8% to 11%, while patients’ visits to GPs and using services including telehealth, email, and mHealth, increased substantially [30].

The calculations in our study are based solely on public expenditure and utilisation by outpatients, excluding data from hospitals. We could not evaluate whether the patients switched between INNs due to an aggregated level of collected data. Another limitation is the retrospective approach towards the data collection and processing that might lead to an underestimation of the utilisation data. The absence of DDDs of fixed dose combinations can also be considered as a limitation. Based on the WHO recommendations we used the dose of 1 tablet for FDC as DDD. In the case of FDCs with some different dosage options, an average of all available dosage forms was used.

## 5. Conclusions

Analysis of the penetration of antidiabetic medicines on the Bulgarian market, their utilisation, and reimbursed expenditures pre-, post-, and during the pandemic period provide valuable insights into the availability of antidiabetic medicines to patients. We found an increase in utilisation and cost paid for antidiabetics. We did not find shortages of antidiabetics and could consider that the COVID-19 pandemic did not affect organisation of the supply and financing of antidiabetic medicines in Bulgaria.

## Figures and Tables

**Table 1:** Reimbursed cost (Euro) and index of change.

INN	2018	2019	2020	2021	Index (Pandemic vs. Pre-Pandemic)	2022	2023	Index (Post-Pandemic vs. Pandemic)
	Pre-Pandemic Period		Pandemic Period			Post-Pandemic Period		
Acarbose	979,285.1	932,910.21	954,207.89	875,388.42	−0.01	799,427.68	747,599.92	−0.04
Canagliflozin	449,121.98	196,341.34	436,374.53	352,532.47	0.06	282,352.88	255,193	−0.08
Canagliflozin/ Metformin	NA	9132.58	66,224.16	54,222.09		42,245.4	47,105.025	−0.06
Dapagliflozin	306,518.88	491,513.55	707,097.98	765,126.3	0.21	2,924,269.23	8,407,953.6	1.67
Dapagliflozin/ Metformin	1,862,607.8	2,393,036.2	3,011,861.3	3,580,888.7	0.14	4,361,382.73	5,136,998.8	0.11
Empagliflozin	523,505.1	1,256,714.7	1,338,573.2	1,312,299.4	0.12	2,076,101.92	1,864,353.9	0.12
Empagliflozin/ Linagliptin	NA	NA	140,037.85	481,164.31		830,924.25	1,485,520.2	0.68
Empagliflozin/ Metformin	1,430,686.1	2,442,768.1	3,242,722.2	3,823,186.7	0.21	4,657,127.48	6,001,389.2	0.13
Gliclazide	3,144,123.6	3,010,073.3	3,547,025.5	3,885,917	0.05	3,897,379.7	4,021,482.34	0.02
Glimepiride	836,541.69	737,046.03	721,634.43	687,456.15	−0.03	783,555.47	866,762.2	0.04
Linagliptin	347,960.54	746,887.71	834,450.62	862,393.03	0.14	861,559.38	926,469.67	0.01
Linagliptin/ Metformin	3,625,868.8	3,708,102.3	3,673,899.	3,521,786.4	0.00	3,337,941.4	3,205,834.90	−0.02
Metformin	3,243,165	3,155,736	2,935,885	2,902,115	−0.02	2,781,089.29	2,639,609.4	−0.02
Pioglitazone	58,584.6	60,511.7	53,447.08	50,477.7	−0.03	63,710.79	110,933.42	0.17
Pioglitazone/ Metformin	NA	4789.73	33,073.67	37,481.26		46,979.88	67,063.39	0.15
Repaglinide	29,643.63	52,573.62	38,693.5	32,076.37	−0.03	28,847.14	24,679.43	−0.06
Saxagliptin/ Metformin	633,477.22	495,938.07	391,976.48	311,995.98	−0.09	248,675.59	196,087.56	−0.09
Saxagliptin/ Dapagliflozine	NA	NA	NA	15,113.11		149,702.97	365,255.87	
Semaglutide	NA	NA	NA	NA		1,145,036.55	12,908,340.6	
Sitagliptin	167,253.45	148,836.34	157,028.63	134,294.9	−0.02	114,454.83	46,478.54	−0.11
Sitagliptin/ Metformin	2,024,628.7	1,675,429	1,446,603.3	1,224,820.2	−0.07	979,262.31	470,475.76	−0.11
Vildagliptine	220,505.77	52,657.45	51,507.06	49,350.42	−0.16	51,428.05	59,934.61	0.03
Vildagliptine/ Metformin HCL	1,816,532.1	1,666,291.1	1,961,351.7	2,169,266.7	0.05	1,820,042.87	1,863,471.23	−0.03
Total costs	21,700,010	23,237,289	25,743,675	27,129,352	0.04	32,283,498	55,500,013	0.17

**Table 2:** Utilisation in DTD and index of changes.

INN	DDD/ 1000 Inh/Day 2018	DDD/ 1000 Inh/Day 2019	DDD/ 1000 Inh/Day 2020	DDD/1000 Inh/Day 2021	Index (Pandemic vs. Pre-Pandemic Period)	DDD/ 1000 Inh/Day 2022	DDD/ 1000 Inh/Day 2023	Index (Post-Pandemic vs. Pandemic Period)
	Pre-Pandemic Period		Pandemic Period			Post-Pandemic Period		
Acarbose	1.071	0.993	1.01	0.93	−0.02	0.9	0.842	−0.03
Canagliflozin	0.002	0.026	0.056	0.048	0.68	0.041	0.038	−0.06
Canagliflozin/ Metformin	NA	0.007	0.048	0.042		0.035	0.039	−0.04
Dapagliflozin	0.167	0.144	0.204	0.223	0.09	1.017	5.666	3.66
Empagliflozin	1.762	2.324	2.935	3.56	0.15	4.726	0.33	−0.06
Empagliflozin/ Linagliptin	0.15	0.199	0.214	0.215	0.06	0.383	0.471	0.25
Empagliflozin/ Metformin			0.035	0.138		0.263	6.58	9.64
Dapagliflozin/ Metformin	1.399	1.49	3.174	3.823	0.36	5.129	5.666	0.14
Gliclazide	14.663	14.189	14.312	14.371	0.00	15.197	16.156	0.02
Glimepiride	8.406	8.302	8.155	7.844	−0.01	8.047	8.16	0.00
Linagliptin	0.172	0.217	0.25	0.274	0.09	0.297	0.32	0.04
Linagliptin/ Metformin HCL	3.627	3.752	3.787	3.741	0.01	3.841	3.715	0.00
Metformin	20.072	19.668	18.213	18.385	−0.02	18.689	17.74	0.00
Pioglitazone	0.261	0.295	0.28	0.265	0.00	0.263	0.264	−0.01
Pioglitazone/ Metformin		0.0204	0.139	0.163		0.169	0.152	0.02
Repaglinide	0.345	0.293	0.219	0.182	−0.09	0.174	0.149	−0.05
Saxagliptin/ Metformin	0.722	0.581	0.469	0.369	−0.09	0.326	0.259	−0.08
Saxagliptin/ Dapagliflozine				0.004		0.044	0.107	
Semaglutide						0.129	1.004	
Sitagliptin	0.079	0.072	0.064	0.0582	−0.05	0.056	0.054	−0.02
Sitagliptin/Metformin	2.428	2.251	2.083	1.861	−0.04	1.721	1.699	−0.03
Vildagliptine	0.024	0.022	0.022	0.02	−0.02	0.024	0.029	0.07
Vildagliptine/Metformin	2.812	2.884	3.331	3.73	0.06	4.394	4.908	0.08
Total annual utilisation	58.16	57.73	59.00	60.25	0.01	65.87	71.78	0.04

## Data Availability

The data that support the findings of this study are available upon reasonable request from the corresponding author (Z.M.).
